# Mutation and Evolutionary Rates in Adélie Penguins from the Antarctic

**DOI:** 10.1371/journal.pgen.1000209

**Published:** 2008-10-03

**Authors:** Craig D. Millar, Andrew Dodd, Jennifer Anderson, Gillian C. Gibb, Peter A. Ritchie, Carlo Baroni, Michael D. Woodhams, Michael D. Hendy, David M. Lambert

**Affiliations:** 1 Allan Wilson Centre for Molecular Ecology and Evolution, School of Biological Sciences, University of Auckland, Auckland, New Zealand; 2 Allan Wilson Centre for Molecular Ecology and Evolution, Institute of Molecular BioSciences, Massey University, Auckland, New Zealand; 3 Dipartmento Scienze della Terra, Università di Pisa, Pisa, Italy; 4 Consiglio Nazionale Ricerche, Centro Studio Geologia Strutturale, Pisa, Italy; 5 Allan Wilson Centre for Molecular Ecology and Evolution, Institute of Fundamental Sciences, Massey University Palmerston North, Palmerston North, New Zealand; Stanford University, United States of America

## Abstract

Precise estimations of molecular rates are fundamental to our understanding of the processes of evolution. In principle, mutation and evolutionary rates for neutral regions of the same species are expected to be equal. However, a number of recent studies have shown that mutation rates estimated from pedigree material are much faster than evolutionary rates measured over longer time periods. To resolve this apparent contradiction, we have examined the hypervariable region (HVR I) of the mitochondrial genome using families of Adélie penguins (*Pygoscelis adeliae*) from the Antarctic. We sequenced 344 bps of the HVR I from penguins comprising 508 families with 915 chicks, together with both their parents. All of the 62 germline heteroplasmies that we detected in mothers were also detected in their offspring, consistent with maternal inheritance. These data give an estimated mutation rate (*μ*) of 0.55 mutations/site/Myrs (HPD 95% confidence interval of 0.29–0.88 mutations/site/Myrs) after accounting for the persistence of these heteroplasmies and the sensitivity of current detection methods. In comparison, the rate of evolution (*k*) of the same HVR I region, determined using DNA sequences from 162 known age sub-fossil bones spanning a 37,000-year period, was 0.86 substitutions/site/Myrs (HPD 95% confidence interval of 0.53 and 1.17). Importantly, the latter rate is not statistically different from our estimate of the mutation rate. These results are in contrast to the view that molecular rates are time dependent.

## Author Summary

Molecular evolutionary theory suggests that for neutral DNA sequences, rates of mutation and evolution should be equal. However, there has been considerable variation in empirical estimates of rates of molecular change in vertebrate animals, even for the same regions of the mitochondrial genome. A difficulty is that evolutionary rates estimated from ancient DNA and short-term mutation rates are not available for the same species. We present data on the rate of mutation of the mitochondrial hypervariable region in Adélie penguins from the Antarctic. All recorded mutations were heteroplasmic in mothers, and almost invariably, both genetic variants were passed to their offspring. We compared this rate of mutation to the rate of evolution estimated using serially preserved ancient remains. We show that these two estimates were not statistically different from each other.

## Introduction

Precise estimates of molecular rates are fundamental to our understanding of the processes of evolution. In principle, mutation and evolutionary rates for neutral regions of the genome from the same species are expected to be equal [1]. However, on the basis of empirical findings, it has recently been argued that molecular rates vary in relation to the time period over which they are measured [2]–[4]. For example, Parson et al. [5], Santos et al. [6] and Howell et al. [7] reported very high mutation rates of the mitochondrial control region using human pedigrees. More recently, a series of ancient DNA-based estimates of evolutionary rates of the control region have been reported in a range of other vertebrate animals [8]–[10]. For example, Lambert et al. [8] recorded a rate of 0.96 substitutions/site/Million years (s/s/Myrs) for the HVRI region of the mitochondrial genome of Adélie penguins. These rates are also typically high, and are similar to many pedigree rate estimates [e.g. 7]. Furthermore, rates estimated using inter-specific divergence levels, typically calibrated against the fossil record [11] or some biogeographic event [12], are slower than the ancient DNA-based estimate. Overall, these rates of molecular change differ by up to an order of magnitude [13].

These recent findings have given rise to the suggestion that the relationship between molecular rates and the times over which they are measured follows an exponential decline, the so-called ‘lazy jay’ curve [2]. Ho et al. [4] suggested that it takes 1–2 Myrs for the rate of molecular change to decline to a constant substitution rate. If this temporal variation in molecular rates is substantiated, it has important theoretical implications as well as practical ones. Specifically, time dependency would require that rates be estimated over short and long time periods for each species or group of species, and then used only for the appropriate time interval. Resolving this apparent difference in rates is difficult because precise estimates of both mutation and evolutionary rates for the same species have not been possible. Also many comparisons have been made using data from different species. For example, Ho et al. [3] compared rates of molecular change estimated from many avian species.

Therefore, in order to compare molecular rates accurately over different time periods and within a single species, the following are required: extant natural populations from which large numbers of pedigree samples can be collected, along with large numbers of ancient samples of the same species from an undisturbed environment. Adélie penguins meet these requirements and therefore represent an ideal model for resolving disparate views about the time dependency of molecular rates. Using this species, it is possible to estimate both mutation and evolutionary rates for the same region of the genome precisely.

In Adélie penguins, breeding birds inhabit ice-free areas of the Antarctic coastline during the summer months. Adult pairs typically lay two eggs, and both adults and chicks can readily be blood sampled and banded. In order to test the time dependency of rates, we estimated the mutation rate by sequencing the HVR I of penguins from a large number of known families comprising both parents and chicks. Adélie penguins are also characterised by the presence, under breeding colonies, of large collections of sub-fossil bones of the same species. Given the typically high level of natal return that is characteristic of this species [14], these remains generally have a close genealogical relationship to the birds now nesting on top of them. In addition, the very cold and dry Antarctic environment has favoured the preservation of both nuclear [15] and mitochondrial DNA [8],[16]. We have expanded a previous dataset and estimated the evolutionary rate for the same HVR I region using a large number of sub-fossil bones aged up to 37,000 yrBP [8],[16]. These studies allowed us to assess the relationship between mutation and evolutionary rates of a single region of the genome in Adélie penguins directly and thereby directly test current ideas about the time dependency of molecular rates.

## Results

**Figure 1:**
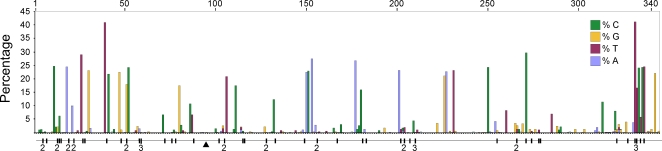
A plot of the frequency of the non-majority nucleotide bases across the 344 bp region of the HVR I for all adult penguins examined in this study. Only individuals that showed no heteroplasmies were used. The sites that showed transitional mutations are shown. When more than one transition was recorded at a site, the number is shown. The position of the single transversional mutation that was recorded is shown (triangle).

**Figure 2:**
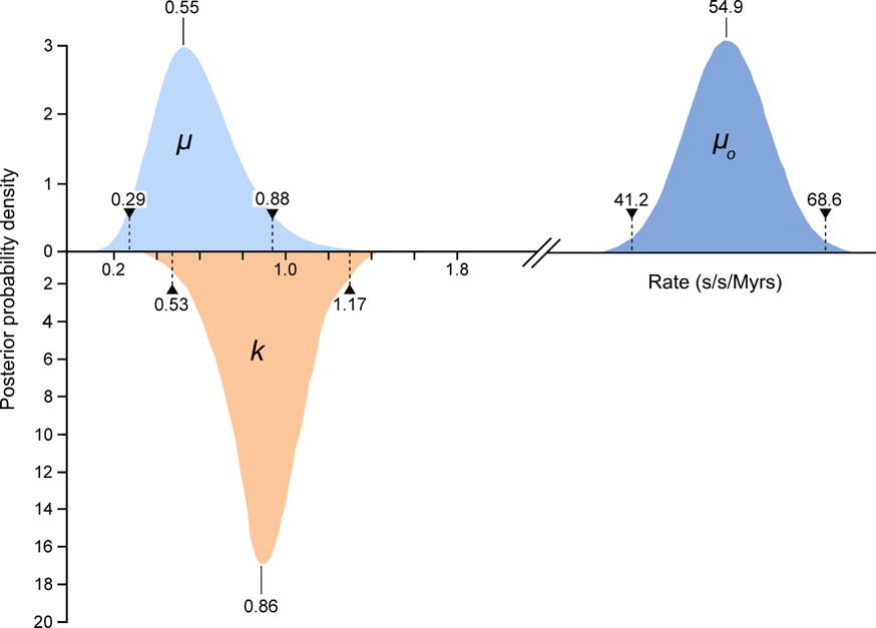
The estimates of the frequency of observed heteroplasmies (*μ*_o_) with the mean and 95% confidence intervals are shown. The distribution, mode and confidence intervals of the mutation rate (*μ*) derived from modeling the inter-generational persistence of the heteroplasmies are also shown. The posterior probability densities of the evolutionary rate (*k*), estimated from 162 ancient penguin bones of known ages, are shown below the line. The median and 95% intervals are given.

We compared high quality DNA sequences from a 344bp region of the 5′ terminus of the mitochondrial HVR I region of a large sample of modern Adélie penguins. These comprised the mother and father from each of 508 families and typically two chicks per family. All blood samples were collected from an Adélie penguin colony at Cape Bird, Ross Island, Antarctica over four consecutive summers starting in 2001/2. DNA sequences were scored for quality using PHRED [17] and poor sequences were eliminated from our analysis or re-sequenced (see Materials and Methods). From the remaining sequence data, a number of mitochondrial heteroplasmic sites were detected. At these sites, two nucleotide signals were apparent in the same individual. Such heteroplasmies are the result of an earlier mutation event and two variants (the original and the mutant) have persisted in the same individual. In order to rule out false positives, such as substitutions that might arise from PCR amplification errors, all heteroplasmies were re-sequenced from different DNA extractions from the same samples. In total, we detected 62 heteroplasmies from DNA trace data. A calibration study (see Materials and Methods) showed that the proportions of each mitochondrial haplotype can be accurately inferred from the DNA trace data, with a standard error of approximately 5%. All but one of the recorded heteroplasmies were transitions and all but three of the heteroplasmic sites were at positions in which polymorphisms were recorded in populations of Adélie penguins from colonies in the Ross Sea, Antarctica. Figure 1 illustrates the position of the heteroplasmic sites and the frequency of the non-majority base in each case. Using available Adélie penguin life history data [14],[18], we estimated the average intergenerational age (g) as 6.46 years. Using these data, the observed rate of heteroplasmies (*μ*_o_) of the HVR I region is 54.9 mutations/site/Myr with 95% confidence intervals of 41.2–68.6 mutations/site/Myr (Figure 2).

Mutation events in mitochondria result in heteroplasmies that can persist over generations and which may or may not be detected, depending on the frequency of rarer variants [19],[20]. In our study, heteroplasmies appeared to be germline variants rather than somatic, as evidenced by the fact that, in all families, they were transmitted from the mother to one or both chicks (Table 1). In combination, these data suggest that, in contrast to mutations in some other species [6],[21], mutations in Adélie penguins persist in the heteroplasmic state for many generations. A heteroplasmy can only be transmitted across generations if a chick inherits multiple copies of its mother's genome. The larger the number of generations that a heteroplasmy persists, the higher will be the probability that it will be detected. A heteroplasmy can persist for many generations in a maternal line of descent until it is either lost or goes to fixation. This persistence time is influenced by the number of segregating mitochondrial genomes (*N*) that pass through the inheritance bottleneck. For human oocytes, *N* has been estimated to be between 15 and 70 [19],[22].

**Table 1:** Details of heteroplasmies recorded from pedigree material of Adélie penguin families.

		Ratio of heteroplasmic sites in each family member		
Variable Nucleotide Site	Heteroplasmy	Mother	Offspring	
57	A/G	55∶45	43.5∶56.5	42.5∶57.5
333	T/C	50∶50	54∶46	62∶38
277	T/C	49∶51	40∶60	46∶54
58	G/A	40.5∶59.5	44∶56	25∶75
**39**	**C/T**	**62∶38**	**55∶45**	**60.5∶39.5**
**330**	**G/A**	**47.5∶52.5**	**39.5∶60.5**	**35.5∶64.5**
206	T/C	49.5∶50.5	59.5∶40.5	54.5∶45.5
27	C/T	70∶30	66.5∶33.5	69∶31
254	G/A	65.5∶34.5	72∶28	72∶28
18	G/A	49∶51	65.5∶34.5	53.5∶46.5
104	C/T	71.5∶28.5	62.5∶37.5	66.5∶33.5
326	A/G	71∶29	31∶64	32.5∶67.5
13	A/G	64∶36	59∶41	52∶48
21	G/A	52.5∶47.5	43∶57	41∶59
**273**	**A/G**	**70∶30**	**58.5∶41.5**	**65.5∶34.5**
**335**	**T/C**	**59.5∶40.5**	**79.5∶20.5**	**47∶53**
203	T/C	68∶32	48∶52	64.5∶35.5
**4**	**T/C**	**59.5∶40.5**	**47.5∶52.5**	**50∶50**
**50**	**G/A**	**47∶53**	**56.5∶43.5**	**48.5∶51.5**
209	T/C	69.5∶30.5	75.5∶24.5	78.5∶21.5^2^
104	C/T	76∶24	71∶29	57.5∶42.5
87	C/T	19∶81	31∶69	17.5∶82.5
75	A/G	71.5∶28.5	72.5∶27.5	73.5∶26.5
26	C/T	65∶35	60.5∶39.5	65.5∶34.5
58	G/A	71∶29	50∶50	60∶40
132	T/C	49.5∶50.5	47.5∶52.5	34∶66
166	A/G	16∶84	28∶72	16.5∶83.5
148	A/G	40∶60	21.5∶78.5	33.5∶66.5
58	A/G	37.5∶62.5	44.5∶55.5	36.5∶63.5
**6**	**T/C**	**66∶43**	**63.5∶36.5**	**68∶32**
**331**	**C/T**	**44.5∶55.5**	**50∶50**	**54∶46**
265	A/G	33∶67	29.5∶70.5	26∶74
12	T/C	37.5∶62.5	49∶51	56.5∶43.5
155	G/A	77.5∶22.5	72∶28	66.5∶33.5
209	T/C	51∶49	50∶50	34∶66
12	T/C	39.5∶60.5	49.5∶50.5	42.5∶57.5
155	G/A	71∶29	73∶27	57.5∶42.5
331	C/T	65.5∶34.5	58.5∶41.5	61∶39
18	G/A	83∶17	65.5∶34.5	73.5∶26.5
77	T/C	52.5∶47.5	61∶39	63.5∶36.5
127	A/G	39∶61	34∶66	47.5∶52.5
278	C/T	46∶54	45∶55	42∶58
**201**	**A/G**	**31.5∶68.5**	**48∶52**	**53.5∶46.5**
**265**	**A/G**	**57∶43**	**70∶30**	**70.5∶29.5**
114	C/T	73∶27	75∶25	52.5∶47.5
71	T/C	38∶62	36∶64	1
47	G/A	35∶65	27.5∶72.5	45.5∶54.5
180	T/C	70.5∶29.5	60∶40	69∶31
270	A/G	74.5∶25.5	75.5∶24.5	92∶8
320	T/C	46∶54	41.5∶58.5	26.5∶73.5
182	G/A	65∶35	74∶26	72.5∶27.5
21	G/A	43.5∶56.5	63∶37	1
209	T/C	58.5∶41.5	47∶53	26∶74
101	C/T	69∶31	68∶32	77∶23
127	A/G	42.5∶57.5	28.5∶71.5	19.5∶80.5
115	T/C	45∶55	35.5∶64.5	31.5∶68.5
14	T/C	71.5∶28.5	63∶37	63∶37
**94**	**T/G**	**54∶46**	**50∶50**	**60.5∶39.5**
**331**	**C/T**	**70.5∶29.5**	**66.5∶33.5**	**66∶34**
203	T/C	60∶40	58.5∶41.5	56.5∶43.5
**4**	**T/C**	**49.5∶50.5**	**50∶50**	**51.5∶48.5**
**50**	**G/A**	**48.5∶51.5**	**41.5∶58.5**	**50∶50**

1 Indicates one chick only in this family.

2 Indicates a third heteroplasmic chick (209 T/C 91.5∶8.5) in this family.

Bold indicates examples of heteroplasmies at two sites in a mother and her chicks.

Our estimate of the mutation rate is affected by our ability to discriminate between low frequency heteroplasmies and noise in DNA trace data. If we set the threshold detection level too low, we would mistakenly include ‘noise’ as evidence of heteroplasmies. To avoid such false positives, at least one of the two chicks had to have a haplotype that exceeded a threshold level. As a result of a calibration study (Materials and Methods), we set a detection threshold frequency (*θ*) of 23%. Our further analyses take account of the expected number of heteroplasmies that are excluded using this threshold.

We used a recently developed model [23] to take account of the above factors in our estimate of the mutation rate. The model defines the rate at which new mutations enter the germ-line (α). Assuming these mutations are neutral and that each mutation is equally likely to be transmitted to the next generation, only 1*/N* of these mutations are expected to go to fixation, so *μ* = α/*N*. This model [23] assumes that we can only observe a heteroplasmy when the proportion of a new haplotype exceeds *θ*. This model also assumes that if *θ* = 0.23, most heteroplasmies are lost without reaching this proportion, and most heteroplasmies that reach this level do not go to fixation. If *N* were doubled, then the number of heteroplasmies that reach the threshold halves, but the observed persistence of those that reach it, doubles. Hence, the rate of observed heteroplasmies (*μ*_o_) is independent of *N*, and approximated by the expression 2α/ln(1/*θ*−1) [22]. Thus for the threshold *θ* = 0.23, *μ*_o_ ≈ 2.417α, so *μ*≈0.414 *μ*_o_/*N*.

**Figure 3:**
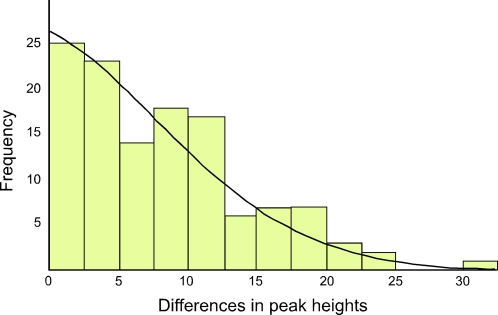
The distribution of differences between the two haplotypes in heteroplasmic mothers and their chicks is shown, as determined by DNA trace peak heights. The curved line represents the prediction of the model, given the estimated number of mitochondria transmitted between mothers and chicks (*N* = 36.5).

The ratio of the heteroplasmic variants present in mothers and chicks was estimated from the relative peak heights in DNA trace data for each individual (Table 1). These represent the relative proportions of each of the four possible nucleotides at any given position in a DNA sequence. Using the binomial distribution to model the inheritance of heteroplasmies, we showed that for a large *N* there is little difference in heteroplasmy ratios between mothers and chicks. We used this finding to infer *N,* from the distribution of differences in heteroplasmy ratios of mothers and chicks. These differences are summarised in Figure 3.

We present here a brief description of the method used to estimate *N*. A more detailed description is found in [23]. From Table 1, we calculated the mean square difference in the frequency of haplotypes (as estimated by the peak heights in DNA trace data) between mothers and chicks, which we designated the “raw variance” (σ ˆ^2^_raw_). The raw variance is dependent on the actual heteroplasmy difference between mother and chick, which we designated the “genetic variance” (*σ* ^2^_genetic_), and the uncertainties in measuring the heteroplasmy, known as the “measurement variance” (*σ* ^2^_measure_). The genetic variance was estimated by subtracting the measurement variance from the raw variance. *N* was estimated from this using the variance of a binomial distribution. We also estimated the uncertainties in our analysis. For a Gaussian distribution, the variance in the sample variance is var (σ ˆ^2^) = (n−1)/n^2^ *σ* ^4^ [24], where *n* is the number of samples and *σ* ^2^ represents the true variance.

Within the data in Table 1, there are 123 mother-chick pairs, with mean square difference σ ˆ^2^_raw_ = 1.15×10^−2^ (corresponding to a root mean square of 10.71%) with estimator variance var (σ ˆ^2^_raw_) = 122/123^2^×(1.15×10^−2^)^2^ = 2.12×10^−6^. From the calibration study, each measurement has variance σ ˆ^2^_measure_ = 2.14×10^−3^ (i.e. a standard error in measurement of 4.62%) with estimator variance var (σ ˆ^2^_measure_) = 3.58×10^−7^. Then the genetic variance is σ ˆ^2^_genetic_ = σ ˆ^2^_raw_−2σ ˆ*^2^*_measure_ = 7.18×10^−3^ with estimator variance var (σ ˆ^2^_genetic_) = var (σ ˆ^2^_raw_)+2 var (σ ˆ^2^_measure_) = 2.83×10^−6^ (corresponding to (8.48±0.99)%). If the proportion of each haplotype inherited by the chick comes from a binomial distribution with population size *N* then *σ^2^*_genetic_ = *p(1−p)/N,* where *p* is the mother's heteroplasmy ratio. The expression *p(1−p)* varies little, so we use the mean value of 0.234 to estimate 1/*N* = 0.0319±0.0075 (standard error), which then becomes *N* = 31.3 (95% confidence interval 21.5–57.9). Using a more detailed model [23], we found that the posterior distribution of *N* had a median value 38.3 and HPD 95% confidence intervals 24.3 to 63.3. Using a maximum likelihood estimation, the corresponding point estimate for *μ* is 0.55 mutations/site/Myrs with a HPD 95% confidence interval of 0.29–0.88 mutations/site/Myrs (Figure 2). A number of authors have recently reported similar high rates of mutation from organisms as phylogenetically diverse as *Caenorhabditis elegans* [25] and humans [5],[6],[7],[26].

In order to compare an evolutionary rate with this mutation rate, we expanded on an earlier study [8],[16] that analysed 96 known age sub-fossil bones of Adélie penguins to determine the evolutionary rate for the HVR I region. For this study, we sequenced an additional 66 bones of ages up to 37,000 years (Tables S1) and estimated the rate of evolution for the same 344 bps of the HVR I region used to estimate the mutation rate. These data were characterised by 156 segregating sites and a nucleotide diversity of 0.049 (±0.006 S.E.). The majority of the substitutions were transitional changes (0.047±0.006 S.E.). We estimated the rate of evolution using a Bayesian Markov chain Monte Carlo (MCMC) approach, as implemented in the software Bayesian Evolutionary Analysis Sampling Trees (BEAST v1.3) [27]. An MCMC simulation of 20 million steps, with the first 500,000 steps discarded as the burn-in time, estimated *k* to be between 0.53 and 1.17 substitutions/site/Myr (95% HPD) with a median value of 0.86 substitutions/site/Myr. Our previous median estimate of *k* was 0.96 substitutions/site/Myr and our new analysis reduced the confidence interval from 0.53 to 1.43 [8]. Our analysis showed that the results were not significantly dependent upon the priors.

In relation to the power of our analysis, if *μ* was, for example, four times as large as we observed, we would have recorded approximately four times as many heteroplasmies. Hence, the relative size of the confidence interval would be reduced by a factor of two (but as *μ* quadruples, the absolute size of the confidence interval would double). Approximating the posterior distributions of *μ* and *k* as normal distributions, we find that for *μ*<0.44 or *μ*>1.44, we could reject the null hypothesis (*μ* = *k*) at the 95% confidence level.

## Discussion

The estimation of rates of molecular change is one of the most important exercises in evolutionary biology. Using a pedigree approach and a recently reported mathematical model, we have estimated the mutation rate for the Adélie penguin hypervariable region of the mitochondrial genome as 0.55 mutations/site/Myr. This rate is likely to represent the best estimate of the real mutation rate of the species, excluding lethal mutations. It is similar to rates estimated for the same hypervariable region for humans [see 6 for a summary]. However, it is widely accepted that a range of factors can contribute to inaccurate estimates of rates of molecular change [28]. In relation to longer term evolutionary rates, these factors range from uncertainty in the timing of calibration points, such as the presence of older undiscovered fossils, incorrect phylogenetic relationships among the forms being studied and imprecise estimates of the levels of genetic divergence. However, estimates of mutation rates have not generally been subject to the same level of scrutiny, stemming from an assumption that mutation rate estimates are inherently more precise. It is now clear that some previous estimates of mutation rates from pedigree data have also been subject to a range of errors, e.g. earlier less sensitive DNA sequencing technologies. In addition, different studies have treated heteroplasmies in different ways, with consequent effects on mutation rates. Some studies have included heteroplasmies in the estimation of mutation rates [e.g. 5], while some have not [e.g. 29]. Others have included them but weighted their contribution to the estimation of rates [6]. Howell [7] estimated a mutation rate for the control region of the human mitochondrial genome assuming that each heteroplasmy contributes to the actual mutation rate. According to our model, this assumption over-estimates *μ* by a factor of 5. Thus the authors assume *μ* = α, using our notation. The authors go on to suggest that theirs is an under-estimate of α. In addition, the unique nature of mitochondrial DNA has not been widely appreciated when calculating mutation rates and we suggest that many of these issues are likely to have contributed to poor estimates of mitochondrial mutation rates. The recently reported model [23] explicitly takes account of the above issues and we suggest such analyses are necessary to estimate mutation rates accurately.

When we take these factors into consideration, our comparative data on molecular rates of change illustrate that for the 5′ end of the mitochondrial HVR I in Adélie penguins, the short-term mutation and longer-term evolutionary rates are similar (Figure 2). The respective confidence intervals overlap and they cannot be distinguished statistically (p = 0.13). Hence, the hypothesis that molecular rates vary over time [2]–[4] cannot be supported and our data suggests that the hypervariable region of the mitochondrial genome is evolving neutrally.

Emerson [30] has recently criticised the reliability of some of the data that underlie the proposed relationship between rate estimates and time. Based on a reanalysis of the studies used to estimate rates, Emerson suggested that, for the mitochondrial control region of vertebrate animals, there is little or no relationship between molecular rates and time (see Figure 5b in 29]. In a response to this critique, Ho et al. [31] not only defended their earlier analysis but, based on a reanalysis of ancient DNA data from bison, argued that there is evidence for time dependency of rates over periods less than 1–2 Myrs (see Figure 1 from [4]). Using a sliding window approach where the authors analysed bison samples from a range of ages, they showed that ancient bison DNA sequences from 0–10,000 yrBP evolved at a rate higher than that recorded for older samples, e.g. 0–50,000 yrBP. These rate distributions are, however, not statistically different from each other and the likely effects of small sample sizes remain unresolved. Emerson [30] also questioned estimates of molecular rates based on ancient DNA and suggested that they may generally be upwardly biased. This could be, for example, due to a reduction in population size over the time period studied. This would result in higher rates of fixation of even deleterious mutations and thereby result in an increase in molecular rates. However, since high molecular rates have been observed in ancient DNA studies of such diverse groups as primates, artiodactyls, rodents and birds [3], such a universal population decline in all these species seems unlikely.

Finally, our data do not support the concept of a time dependency of molecular rates. If there is a decline in molecular rates in Adélie penguins over time, it is not apparent over the 37,000 year time period examined in this study. Ho et al. [31] suggested from their data that such a decline should have been observed over the time period we studied. We suggest that, the differences in rates of mutation and evolution that have been detected in some studies may be due to empirical errors associated with heteroplasmy and/or inaccuracies associated with calibration methods.

## Materials and Methods

### Study Site

**Figure 4:**
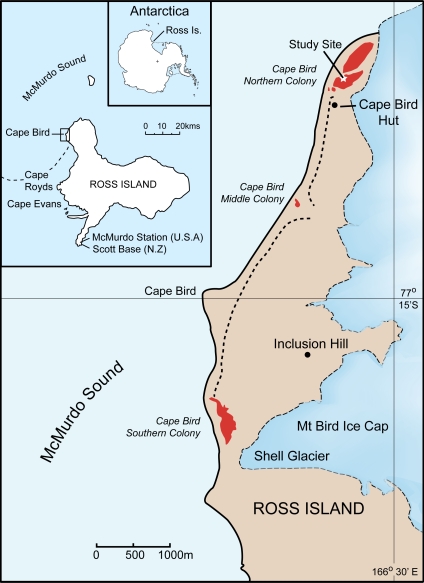
Location of the study site at Cape Bird, Ross Island, Antarctica.

The study was conducted at the Northern Cape Bird Adélie penguin colony, Ross Island, Antarctica (Figure 4). All samples were blood collected within an approximately 1000m area within the colony in order to minimise any disturbance to the colony as a whole. Bleeding protocols were approved by Massey University Animal Ethics Committee permits numbers 00/161 and 03/89. Permission to restrain, take blood and work in the Cape Bird Adélie penguins colony was given by Antarctica New Zealand permit numbers 00/007, 01/007, 02/007, 03/030 and 04/030. Blood sampling was typically conducted from the middle of December, soon after the first eggs had hatched, to early January. During this period, the chicks never leave the nest and are always attended by an adult bird. The parents take turns at guarding the chicks and change over every one to three days [14],[18]. During this period, the adult away from the nest feeds at sea. On his/her return to the nest, there is a ritualistic exchange that consists of highly visible greeting calls before the birds change roles and the sitting bird leaves to feed at sea. Family groups were initially identified by the presence of two adult birds and two chicks at a nest site. The adult bird behaviour was then observed; two birds were only considered to be a pair if they were seen to exchange greetings calls and defend the nest when approached. Once family groups had been identified, the nest location was marked, and the adults and chicks removed from the nest. Adults were restrained with a hand net or shepherd's crook, and chicks were caught by hand. The adult birds were placed in a bag and weighed using either a 5 or 10kg Pesola balance. Adult Adélie penguins were permanently marked using a flipper band. These bands had numerals that can be read easily using 8–10 X binoculars from a distance of 10m. Chicks were weighted using either a 1kg or 0.5kg Pesola balance. Typically, 100ml blood samples were taken from the intertarsal vein of adults, using a 1ml syringe with a 25 gauge needle, after the leg had been cleaned with 70% ethanol. Each syringe was washed with 0.2M di-potassium EDTA, preventing the blood from coagulating. The samples were subsequently stored in pre-labelled tubes containing 1 ml of preservation buffer [32] and stored at 4°C on return to the laboratory. While the adults were bled, the chicks were kept confined to a small bag for warmth and protection. Chicks were similarly bled; however, blood was generally collected from the foot intra-digital vein using a 0.5ml syringe with a 29 gauge needle. All birds were returned to the nest and the temporary nest marker removed. Nests were then observed to ensure that one of the adults resumed guarding the chick. Typically, only pairs with two chicks were sampled. The collection dates and samples used in the study are as follows: from the 1993/4 and 1994/5 breeding seasons, there were five nests comprising 25 individuals; in the 2001/2 breeding season, there were 181 nests comprising 537 individuals; in the 2002/3 breeding season, there were 223 nests comprising 881 individuals; in the 2003/4 breeding season there were 132 nests comprising 525 individuals and finally, in the 2004/5 breeding season, there were 170 nests comprising 650 individuals. In total 2618 individuals from 711 families were collected.

### Molecular Methods

DNA was extracted from the blood/Queens buffer mixture using a Chelex-based method [33]. For each sample, 10 µl of blood was mixed with 80 µl of 10% w/v Chelex (Biorad) in water and heated to 90°C for 25 mins in a thermal cycler. After allowing the sample to cool to room temperature, the sample was centrifuged for 1 min at 16,000g in a microcentrifuge. For each amplification reaction, 1 µl of supernatant was used as template. Polymerase Chain Reaction (PCR) amplifications were carried out in 25 µl volumes containing 1 µl of Chelex-extracted DNA, 1U of Platinum Taq (Invitrogen), 1.5 mM MgCl_2_, 2 µg/µl bovine serum albumin, 0.4 µM of each primer and 200 µM of each dNTP. Samples were amplified using a Applied Biosystems 9700 thermal cycler at 94°C for 10 sec, 50°C for 10sec and 72°C for 25 sec for 35–40 cycles. The HVRI region of the mitochrondrial genome was amplified using primers specific to Adélie penguins AH530 (5′- CTGATTTCACGTGAGGAGACCG-3′) and L-tRNA^Glu^ (5′-CCCGCTTGGCTTYTC TCCAAGGTC-3′) (reference numbers correspond to the sequence deposited in GenBank, accession no. AF272143). All PCR products were purified using either PCR product purification columns (Qiagen) or magnetic beads (Agencourt). PCR products were directly sequenced using the ABI PRISM BigDye Terminator Cycle Sequencing Kit (Applied Biosystems) and analysed on either an ABI 377 automated sequencer or an ABI 3730 automated DNA sequencer. All sequences were analyzed using the computer program PHRED (CodonCode, Dedham, MA). After base calling, PHRED assigns a quality value to each base call ranging from 0 to 60, with higher values corresponding to higher sequence quality. The quality values are logarithmically linked to error probabilities, e.g. a PHRED quality score of 10 corresponds to an accuracy of the base call of 90%, whereas a PHRED quality score of 50 matches a correctness of the base call of 99.999%. In this study, sequences were accepted if ≥90% of the bases had quality scores over 20. Sequence alignment and mutation identification was performed using the Sequencher package (GeneCodes, Ann Arbor, MI). Mixed base calling was set in Sequencher at a detection threshold of 30%. Sequencher defines this in terms of the lower peak being at least 30% of the height of the higher, to be scored as a potential mixed base position. In this study, we have defined the detection threshold for mixed bases in terms of the ratio of absolute peak heights to the total signal. The Sequencher threshold then becomes a threshold of 23% under this definition. This threshold was chosen as an appropriate detection threshold to minimise the chance of false positives in the data.

We examined this threshold by performing a calibration study to test the accuracy of detecting heteroplasmic bases in this region of HVR I empirically. Two different Adélie penguin haplotypes were chosen that differ at 28 positions within the 344bp region of HVR I considered in this study. The HVR I region was amplified from each of these samples using the primers AH-530 and L-tRNA^Glu^. After purification, each PCR product was ligated into the vector pCR 2.1 (Invitrogen) and sequenced. Each haplotype clone was linearised and the concentration determined using spectroscopy. Haplotype mixes representing heteroplasmy at different haplotype ratios were generated by mixing the two haplotype clones (Table S2). These samples were amplified and sequenced as described previously. The trace data from each of these haplotype mixes were analysed and our ability to discriminate the heteroplasmic positions accurately was tested. To do this, at each heteroplasmic site, we plotted the expected haplotype ratios against the observed ratios (Figure S1). At each heteroplasmic position, the value should be 1 if the observed ratio is the same as the expected. If the value is >1 or <1, this represents an under-estimate and an over-estimate of the ratio, respectively.

These data show a clear position dependency of each site to estimate the original haplotype ratio accurately, with some sites consistently over-estimating or under-estimating the ratio within each of the mixed samples. From this analysis, we calculated that there is approximately a 5% error in determining haplotype ratios based on a single heteroplasmic position from trace data.

Background noise in DNA trace data is a significant factor when detecting heteroplasmic bases. The variability of peak heights and noise in trace data results in a different level of sensitivity for heteroplasmy detection at different positions. To test the variability of noise within and between sequences, we extracted the noise profile from ten independent sequences for each of the test samples. We determined the sequence of the noise in each sample and aligned these sequences. This revealed that the noise sequence was conserved between samples with 68% identity. A comparison of the noise profiles for each sequence suggested that the noise profile is highly conserved between sequences (data not shown).

The accuracy of detecting heteroplasmic bases is dependent on the noise profile of each sequence. The position of the peak noise is important, as this represents the first site that could be called a false positive. We compared the height of the minor peak at each of the 28 heteroplasmic positions to the peak noise in each of the haplotype mixes. In each haplotype mix, the number of heteroplasmic bases above and below the peak noise threshold were scored (Table S2). From these data, it is clear that at the lower haplotype ratios (10∶90 and 20∶80) a number of the heteroplasmic bases are lower than the noise threshold. Even with the 30∶70, samples not all the heteroplasmic positions can be discriminated from the noise. This finding reduces the sensitivity of heteroplasmy detection and indicates that setting a low detection threshold in an attempt to increase the sensitivity of heteroplasmy detection will also result in an increased likelihood of scoring false positives. These data suggest a detection threshold between 20 and 30% is the most appropriate to maximise sensitivity while, at the same time, minimising the chance of false positives.

Next we tested the ability of the program Sequencher to detect the heteroplasmic positions accurately within each haplotype mix at different threshold settings. The trace data from each haplotype mix were analysed for secondary peaks at three different detection thresholds using Sequencher (Table S3). In each experiment, the ability to detect the heteroplasmic positions for different haplotype ratios with different detection thresholds was tested, the number of correct bases were determined and false positives were scored (Table S3).

This experiment demonstrates that when the threshold is reduced below 23%, for the sequences in this study, the number of heteroplasmic positions detected increases slightly but false positives are included. This supports the use of a detection threshold of 23% for these sequences as a threshold that maximises accuracy and minimises false positives. Table 1 provides details of the level of heteroplasmy in mothers and chicks, as estimated by the peak heights in DNA trace data.

### Evolutionary Rate Estimation

Summary statistics were estimated using Molecular Evolutionary Genetics Analysis (MEGA) v3.1 [34]. The number of variable sites was 156. There were 104 informative sites and 52 singletons. The base composition of the region was as follows: (%T) 31.1, (%C) 18.7, (%A) 30.5, (%G) 19.7. The average pair-wise distance between the sequences was 0.049 (±0.006) per site; the transition and transversion substitutions were 0.047 (±0.006) and 0.002 (±0.000) per site respectively. The evolutionary rate was estimated using Bayesian Evolutionary Analysis Sampling Trees (BEAST) v1.3 [27] with 20 million steps in an MCMC simulation (500,000 step burn-in time). When running an MCMC chain, the number of independent observations is less than the total length. The chain is not random in tree space and the information in each observation is not totally separate from the information in other observations. BEAST circumvents this problem of autocorrelation by adjusting the sample size. The effective sample size is calculated in BEAST as the total chain length divided by the chain autocorrelation coefficient (both metrics are produced by BEAST). Hence, the ESS is 19.5 million/182243.0 = 107.

## Supporting Information

Figure S1 Position dependency of heteroplasmy ratios. Combined plot of expected versus observed heteroplasmy ratios for haplotype mixes, as shown by different colours.
(25.55 MB EPS)

Table S1 Details of sub-fossil bones of Adélie penguins used to estimate the evolutionary rate for the mitochondrial HVR I region, in addition to those detailed previously [8]. A total of 62 collection sites and radiocarbon dates of penguin remains from Victoria Land, Antarctica, are listed. A total of 66 bones were obtained from these sites. Carbon-14 dates were supplied by: Geochron Lab.-Krurger Enterprise Inc., Cambridge Massachusetts (conventional and AMS, GX-); IsoTrace Radiocarbon Lab., Toronto (AMS, TO-); NOSAMS, Woods Hole Oceanographic Institution (AMS, OS-); The Institute of Geological and Nuclear Sciences, Lower Hutt, NZ (AMS, NZA-).
(0.15 MB DOC)

Table S2 Discrimination of heteroplasmic bases from background noise.
(0.04 MB DOC)

Table S3 Threshold accuracy.
(0.05 MB DOC)
