# Supplementary material for: Mutation and Evolutionary Rates in Adélie Penguins from the Antarctic
Source: PLoS Genet. 2008 Oct 3;4(10):e1000209. doi: 10.1371/journal.pgen.1000209 (PMC2546446; doi:10.1371/journal.pgen.1000209)
Supplement: Table S1 — Details of sub-fossil bones of Adélie penguins used to estimate the evolutionary rate for the mitochondrial HVR I region, in addition to those detailed previously [8]. A total of 62 collection sites and radiocarbon dates of penguin remains from Victoria Land, Antarctica, are listed. A total of 66 bones were obtained from these sites. Carbon-14 dates were supplied by: Geochron Lab.-Krurger Enterprise Inc., Cambridge Massachusetts (conventional and AMS, GX-); IsoTrace Radiocarbon Lab., Toronto (AMS, TO-); NOSAMS, Woods Hole Oceanographic Institution (AMS, OS-); The Institute of Geological and Nuclear Sciences, Lower Hutt, NZ (AMS, NZA-). (0.15 MB DOC) [file pgen.1000209.s002.doc]

**Table S1. Details of sub-fossil bones of Adélie penguins used to estimate the evolutionary rate for the mitochondrial HVR I region, in addition to those detailed previously (Lambert et al., 2002). A total of 62 collection sites and radiocarbon dates of penguin remains from Victoria Land, Antarctica are listed. A total of 66 bones were obtained from these sites. ^14^C dates were supplied by: Geochron Lab.-Krurger Enterprise Inc., Cambridge Massachusetts (conventional and AMS, GX-); IsoTrace Radiocarbon Lab., Toronto (AMS, TO-); NOSAMS, Woods Hole Oceanographic Institution (AMS, OS-); The Institute of Geological and Nuclear Sciences, Lower Hutt, NZ (AMS, NZA-).**

|  |  |  |  |  |
| --- | --- | --- | --- | --- |
| **Collection Sites** | **Lat.-Long.** | **Code**  **(collection/lab)** | **Radiocarbon Age**  **(yr BP)** | **Calibrated age**  **(yr BP)** |
|  |  |  |  |  |
|  |  |  |  |  |
| Inexpressible Island | 74°53’S, 163°45’E | NZA-13221 | 3340 ± 85 | 2328 |
| Inexpressible Island | 74°53’S, 163°45’E | NZA-13223 | 3890 ± 80 | 2988 |
| Inexpressible Island | 74°53’S, 163°45’E | NZA-13224 | 3675 ± 90 | 2746 |
| Inexpressible Island | 74°53’S, 163°45’E | NZA-13225 | 2900 ± 90 | 1814 |
| Inexpressible Island | 74°53’S, 163°45’E | NZA-13226 | 3010 ± 220 | 1927 |
| Inexpressible Island | 74°53’S, 163°45’E | NZA-13227 | 5360 ± 90 | 4858 |
| Inexpressible Island | 74°53’S, 163°45’E | NZA-13228 | 5945 ± 340 | 5599 |
| Inexpressible Island | 74°53’S, 163°45’E | NZA-13229 | 1845 ± 75 | 686 |
| Inexpressible Island | 74°53’S, 163°45’E | NZA-13230 | 1860 ± 75 | 702 |
| Inexpressible Island | 74°53’S, 163°45’E | NZA-13231 | 1910 ± 75 | 750 |
| Inexpressible Island | S74°54'36.4'', E 163°42'33.1'' | NZA-13232 | 4570 ± 90 | 3836 |
| Inexpressible Island | S74°54'36.4'', E 163°42'33.1'' | NZA-13233 | 3955 ± 155 | 3091 |
| Inexpressible Island | S74°54'36.4'', E 163°42'33.1'' | NZA-13234 | 4150 ± 60 | 3341 |
| Inexpressible Island | S74°54'36.4'', E 163°42'33.1'' | NZA-13235 | 1910 ± 75 | 750 |
| Terra Nova Station | S74°42'35.1" E164°06'24.5" | 860209.01  GX-12760 | 5770 ± 60 | 5448 |
| Terra Nova Station | S74°42'35.1" E164°06'24.5" | 890204.106  GX-15494 | 4585 ± 105 | 3858 |
| Terra Nova Station | S74°42'35.1" E164°06'24.5" | 890204.107  GX-15495 | 4915 ± 105 | 4339 |
| Terra Nova Station | S74°42'35.1" E164°06'24.5" | 941122.02  TO-5573 | 5100 ± 70 | 4543 |
| Terra Nova Station | S74°42'35.1" E164°06'24.5" | 941122.05  GX-21408 | 5307 ± 310 | 4826 |
| Terra Nova Station | S74°42'35.1" E164°06'24.5" | 970115.01  GX-25109am | 4880 ± 40 | 4270 |
| Terra Nova Station | S74°42'35.1" E164°06'24.5" | 970115.05  GX-25110am | 5040 ± 50 | 4489 |
| Terra Nova Station | S74°42'35.1" E164°06'24.5" | 970115.07  OS-27674 | 6480 ± 45 | 6201 |
| Terra Nova Station | S74°42'35.1" E164°06'24.5" | 941102.13  GX-21406 | 4205 ± 150 | 3385 |
| Terra Nova Station | S74°42'35.1" E164°06'24.5" | 941112.01  GX-20574 | 4625 ± 120 | 3906 |
| Terra Nova Station | S74°42'35.1" E164°06'24.5" | 941102.04  GX-21404 | 5825 ± 125 | 5488 |
| Terra Nova Station | S74°42'35.1" E164°06'24.5" | 961216.01  GX-23275am | 4520 ± 65 | 3801 |
| Terra Nova Station | S74°42'35.1" E164°06'24.5" | 961216.04  GX-23276am | 5310 ± 60 | 4828 |
| Terra Nova Station | S74°42'35.1" E164°06'24.5" | 870119.03  GX-13620 | 4615 ± 85 | 3894 |
| Terra Nova Station | S74°42'35.1" E164°06'24.5" | 941111.01  TO-4964 | 4670 ± 80 | 3970 |
| Terra Nova Station | S74°42'35.1" E164°06'24.5" | 941111.03  TO-4965 | 9220 ± 100 | 8977 |
| Terra Nova Station | S74°42'35.1" E164°06'24.5" | 901217.01  GX-16933 | 1845 ± 75 | 686 |
| Terra Nova Station | S74°42'35.1" E164°06'24.5" | 901217.02  GX-16932 | 1860 ± 75 | 702 |
| Terra Nova Station | S74°42'35.1" E164°06'24.5" | 901217.03  GX-16931 | 1910 ± 75 | 750 |
| Inexpressible Island | 74o53'42''S, 163o42'28''E | NZA-10306 | 4190 ± 80 | 3373 |
| Inexpressible Island | 74o53'33''S, 163o43'43''E | NZA-10306 | 4190 ± 80 | 3373 |
| Inexpressible Island | 74o53'33''S, 163o43'43''E | NZA-10306 | 4190 ± 80 | 3373 |
| Inexpressible Island | 74o53'33''S, 163o43'43''E | NZA-10306 | 4190 ± 80 | 3373 |
| Inexpressible Island | 74o53'33''S, 163o43'43''E | NZA-10306 | 4190 ± 80 | 3373 |
| Inexpressible Island | 74o53'33''S, 163o43'43''E | NZA-10306 | 4190 ± 80 | 3373 |
| Inexpressible Island | 74o53'33''S, 163o43'43''E | NZA-10306 | 4190 ± 80 | 3373 |
| Inexpressible Island | 74o53'33''S, 163o43'43''E | NZA-10306 | 4190 ± 80 | 3373 |
| Inexpressible Island | 74o53'33''S, 163o43'43''E | NZA-10306 | 4190 ± 80 | 3373 |
| Inexpressible Island | 74o53'33''S, 163o43'43''E | NZA-10306 | 4190 ± 80 | 3373 |
| Inexpressible Island | 74o53'33''S, 163o43'43''E | NZA-10306 | 4190 ± 80 | 3373 |
| Inexpressible Island | 74o53'33''S, 163o43'43''E | NZA-10306 | 4190 ± 80 | 3373 |
| Inexpressible Island | 74o53'33''S, 163o43'43''E | NZA-10306 | 4190 ± 80 | 3373 |
| Inexpressible Island | 74o53'33''S, 163o43'43''E | NZA-10306 | 4190 ± 80 | 3373 |
| Inexpressible Island | 74o53'33''S, 163o43'43''E | NZA-12287 | 4150 ± 60 | 3341 |
| Inexpressible Island | 74o53'33''S, 163o43'43''E | NZA-12287 | 4150 ± 60 | 3341 |
| Inexpressible Island | 74o53'33''S, 163o43'43''E | NZA-12287 | 4150 ± 60 | 3341 |
| Inexpressible Island | 74o53'33''S, 163o43'43''E | NZA-12287 | 4150 ± 60 | 3341 |
| Inexpressible Island | 74o53'33''S, 163o43'43''E | NZA-12288 | 3690 ± 70 | 2754 |
| Inexpressible Island | 74o53'33''S, 163o43'43''E | NZA-12288 | 3690 ± 70 | 2754 |
| Inexpressible Island | 74o53'33''S, 163o43'43''E | NZA-12288 | 3690 ± 70 | 2754 |
| Inexpressible Island | 74o53'33''S, 163o43'43''E | NZA-12288 | 3690 ± 70 | 2754 |
| Inexpressible Island | 74o53'33''S, 163o43'43''E | NZA-12288 | 3690 ± 70 | 2754 |
| Inexpressible Island | 74o53'33''S, 163o43'43''E | NZA-12288 | 3690 ± 70 | 2754 |
| Inexpressible Island | 74o53'33''S, 163o43'43''E | NZA-12288 | 3690 ± 70 | 2754 |
| Inexpressible Island | 74o53'33''S, 163o43'43''E | NZA-12288 | 3690 ± 70 | 2754 |
| Inexpressible Island | 74o53'33''S, 163o43'43''E | NZA-12288 | 3690 ± 70 | 2754 |
| Inexpressible Island | 74o53'33''S, 163o43'43''E | NZA-12288 | 3690 ± 70 | 2754 |
| Cape Ross | 76° 43' 59" S - 162° 59' 50" E, ca 30 m | AA-42214- | 30150 +/- 430 | 30150 |
| Cape Ross | 76° 43' 59" S - 162° 59' 50" E, ca 30 m | AA-42218- | 37,570 +/- 940 | 37,570 |
|  |  |  |  |  |
