# Supplementary material for: Mutation and Evolutionary Rates in Adélie Penguins from the Antarctic
Source: PLoS Genet. 2008 Oct 3;4(10):e1000209. doi: 10.1371/journal.pgen.1000209 (PMC2546446; doi:10.1371/journal.pgen.1000209)
Supplement: Table S2 — Discrimination of heteroplasmic bases from background noise. (0.04 MB DOC) [file pgen.1000209.s003.doc]

Table S2. Discrimination of heteroplasmic bases from background noise

| Haplotype ratio  (551:552) | Peak noise* | Mean heteroplasmic  base height* | % of bases  > background noise | % of base  < background noise |
| --- | --- | --- | --- | --- |
| 10:90 | 177 | 109 | 4 | 96 |
| 20:80 | 181 | 183 | 46 | 54 |
| 30:70 | 199 | 299 | 96 | 4 |
| 40:60 | 188 | 419 | 100 | 0 |
| 50:50 | 200 | 523 | 100 | 0 |
| 60:40 | 218 | 514 | 100 | 0 |
| 70:30 | 209 | 385 | 96 | 4 |
| 80:20 | 193 | 237 | 79 | 21 |
| 90:10 | 207 | 130 | 0 | 100 |

*Peak noise and base heights were calculated directly from trace data obtained from scf_dump (Staden package).
