# Supplementary material for: Mutation and Evolutionary Rates in Adélie Penguins from the Antarctic
Source: PLoS Genet. 2008 Oct 3;4(10):e1000209. doi: 10.1371/journal.pgen.1000209 (PMC2546446; doi:10.1371/journal.pgen.1000209)
Supplement: Table S3 — Threshold accuracy. (0.05 MB DOC) [file pgen.1000209.s004.doc]

#### Table S3. Threshold accuracy

| Haplotype ratio  (551:552) | Threshold 16.6%* | | Threshold 20%* | | Threshold 23%* | |
| --- | --- | --- | --- | --- | --- | --- |
|  | % Determined correctly | Number of false positives | % Determined correctly | Number of false positives | % Determined correctly | Number of false positives |
| 10:90 | 0% | 5 | 0% | 0 | 0% | 0 |
| 20:80 | 71% | 3 | 36% | 0 | 14% | 0 |
| 30:70 | 96% | 3 | 93% | 0 | 75% | 0 |
| 40:60 | 100% | 2 | 100% | 0 | 100% | 0 |
| 50:50 | 100% | 5 | 100% | 3 | 100% | 0 |
| 60:40 | 100% | 4 | 100% | 1 | 100% | 0 |
| 70:30 | 89% | 3 | 89% | 1 | 89% | 0 |
| 80:20 | 57% | 5 | 54% | 1 | 43% | 0 |
| 90:10 | 4% | 4 | 4% | 2 | 0% | 0 |
|  |  |  |  |  |  |  |

*These threshold values translate into 20%, 25%, and 30% threshold values respectively as defined in Sequencher.
